# Prospects for probiotics in social bees

**DOI:** 10.1098/rstb.2021.0156

**Published:** 2022-06-20

**Authors:** Erick V. S. Motta, J. Elijah Powell, Sean P. Leonard, Nancy A. Moran

**Affiliations:** Department of Integrative Biology, University of Texas at Austin, Austin, TX 78712, USA

**Keywords:** apiculture, foulbrood, *Nosema*, *Snodgrassella*, *Gilliamella*, microbiome engraftment

## Abstract

Social corbiculate bees are major pollinators. They have characteristic bacterial microbiomes associated with their hives and their guts. In honeybees and bumblebees, worker guts contain a microbiome composed of distinctive bacterial taxa shown to benefit hosts. These benefits include stimulating immune and metabolic pathways, digesting or detoxifying food, and defending against pathogens and parasites. Stressors including toxins and poor nutrition disrupt the microbiome and increase susceptibility to opportunistic pathogens. Administering probiotic bacterial strains may improve the health of individual bees and of hives, and several commercial probiotics are available for bees. However, evidence for probiotic benefits is lacking or mixed. Most bacterial species used in commercial probiotics are not native to bee guts. We present new experimental results showing that cultured strains of native bee gut bacteria colonize robustly while bacteria in a commercial probiotic do not establish in bee guts. A defined community of native bee gut bacteria resembles unperturbed native gut communities in its activation of genes for immunity and metabolism in worker bees. Although many questions remain unanswered, the development of natural probiotics for honeybees, or for commercially managed bumblebees, is a promising direction for protecting the health of managed bee colonies.

This article is part of the theme issue ‘Natural processes influencing pollinator health: from chemistry to landscapes’.

## 1. Introduction

Animal-associated microbial communities often benefit their hosts, and hosts therefore benefit from efforts to protect microbiomes, by limiting adverse impacts of toxins or poor nutrition. In addition to protecting native microbiomes, host health might be enhanced through the application of beneficial, live microorganisms, that is, probiotics [1]. In this article, we explore using probiotics to bolster the health of managed colonies of honeybees and bumblebees. We first review work on the naturally occurring microbial communities associated with social bees, including the considerable evidence on beneficial effects of the native adult gut microbiota. We then summarize investigations on the potential for probiotics in honeybees, with emphasis on probiotics composed of bacterial strains native to honeybee guts. A major unanswered question is whether probiotics establish and persist in recipient hosts. We present new experimental results showing that non-native bacterial strains from a commercial probiotic product fail to establish in the worker bee gut, but a mixture of native gut bacterial strains colonizes robustly and resembles a natural microbiota in eliciting expression of bee genes related to immunity and metabolism. Finally, we discuss future prospects for probiotics in managed bee colonies, including natural strains isolated from bees and natural strains genetically engineered to protect bees.

## 2. Background on microbiomes of social corbiculate bees

Recent research has revealed that naturally occurring microbiomes of social corbiculate bees which include honeybees (genus *Apis*), stingless bees (tribe Meliponini) and bumblebees (genus *Bombus*) are distinctive, have evolved long term with hosts and play positive roles in host health. We summarize these studies and refer readers to other reviews for more details [2–4].

### (a) Adult worker gut microbiomes

Most work has focused on the distinctive communities in guts of workers of the western honeybee *Apis mellifera*. These communities are dominated by five to eight bee-restricted bacterial lineages [5,6]. Bumblebees and stingless bees harbour communities composed of closely related bacterial lineages, with some exceptions in stingless bee groups that have lost particular gut bacterial lineages [6,7].

**Figure 1.**
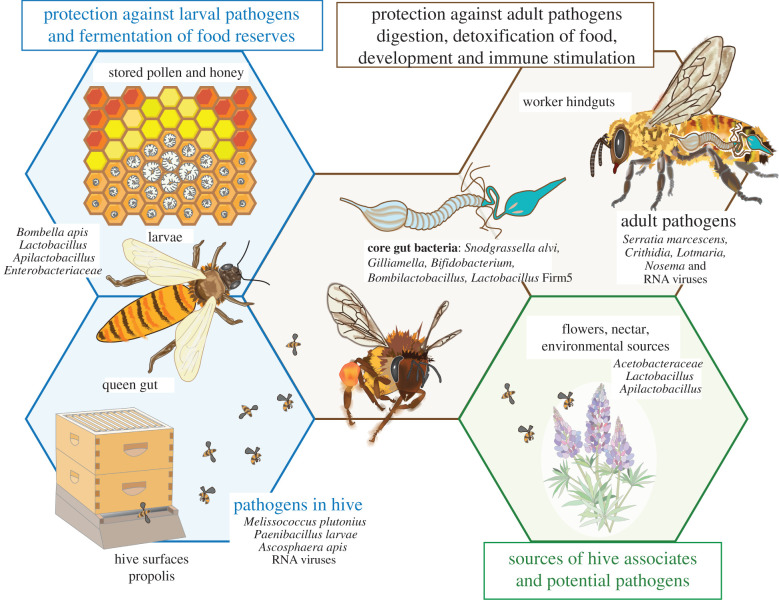
Microorganisms associated with honeybees and their hives. Distinct microbial communities occupy different niches, including a distinct community in hindguts of adult worker bees and a set of microbes exchanged between hive surfaces, food reserves, larval guts, queen guts and worker foreguts. These communities have been shown to confer benefits for bees and hives, but they also include pathogens of larvae and/or adults. Some of these microbes are acquired from or exchanged at environmental sources including nectar and plant surfaces.

In *Apis* and *Bombus*, the main bacterial gut symbionts are acquired via social contact within colonies. *Apis mellifera* workers are colonized soon after emergence as adults, through contact with other workers [8]. The transmitted bacteria grow to a stable community, with a characteristic composition and size, of about 10^8^ cells per worker [9], with similar numbers in bumblebees [6]. Each bacterial species has a characteristic distribution in the ileum or rectum of the hindgut [5,9] (figure 1).

For both honeybees and bumblebees, native gut communities have been shown to support adult worker development and health [2,3]. In experiments that compared workers with or without a normal gut microbiota, the former enjoy a range of benefits that include protection against bacterial, viral, and eukaryotic pathogens [10,11], digestion of components of pollen cell walls [12,13], microbial detoxification of certain sugars [14], stimulation of insulin signalling, appetite, gut development, and weight gain [15] and enhanced production of bee-encoded P450 enzymes that can neutralize dietary toxins [16], although the gut microbiota does not protect against widely used insecticides [17]. In bumblebees, the gut microbiota appears to protect against selenate toxicity [18]. Most experiments in bumblebees (*Bombus terrestris* and *Bombus impatiens*) have focused on protection against parasites and have repeatedly shown that the gut microbiota can defend against trypanosomatid parasites [19–21].

The most widely documented benefit of the adult gut microbiota is protection against invasion by pathogens and parasites. Such protection called ‘colonization resistance’ is a feature of mammalian gut communities [22]. This protective effect may partly reflect enhanced bee immune responses, as the gut microbiota stimulates immune pathways [11,23,24]. However, direct microbe–microbe interactions also appear to contribute, as individual strains vary in protective capacity [20] and possess varying mechanisms for inter-bacterial antagonism [25]. The establishment of biofilms within bee guts also may impose a physical barrier to pathogenic invasion [26,27].

While most evidence for beneficial effects of the bee microbiota comes from laboratory-based studies, experimental and observational evidence supports similar effects in field colonies and populations. In bumblebee populations, microbiota-conferred protection against parasites is supported by lower parasite incidence in individuals with an intact gut microbiota [27]*.* In honeybees, exposure to antibiotic chemicals disrupts the microbiota and lowers worker survivorship both within hives and following laboratory challenge with opportunistic bacterial or eukaryotic pathogens [16,28–31].

### (b) Queen, larval and hive-associated microbiomes

Honeybee queens have gut communities drastically different from those of workers; they lack the core species present in workers, are more erratic in composition and size, and largely consist of environmentally widespread bacterial species [32–34]. By contrast, bumblebee queens have a gut microbiota similar to that of workers and dominated by the characteristic bacterial lineages found in guts of adult corbiculate bees broadly [35].

Microbial communities are also associated with larvae, food reserves and hive surfaces, and these may also affect vigour of the overall colony. In honeybees, all of these communities differ dramatically from those found in adult worker guts [36]. One of the most abundant bacterial species associated with honeybee larvae is a cluster of Acetobacteraceae initially recovered at very low abundance from adult workers [37,38] and later found to be frequent in larval guts and queen guts [32–34,39]. It was initially called ‘Alpha 2.2’, then described as *Parasaccharibacter apium* [36] and more recently placed in its own genus as *Bombella apis* [40]. Several strains isolated from larvae are able to persist in larvae, royal jelly, hypopharyngeal glands and nurse crops [36].

Host-associated communities also can include organisms that are deleterious for hosts [41]. In honeybees, these include viruses such as deformed wing vrus and others [42], bacterial pathogens of larvae such as American and European foulbrood (*Paenibacillus larvae* and *Melissococcus plutonius*) [43], microsporidians in the genus *Nosema* [44], trypanosomatids including *Crithidia* [45] and *Lotmaria* [45]*,* fungal pathogens such as chalkbrood (*Ascosphaera apis*) [46] and opportunistic bacterial pathogens such as *Serratia marcescens* [47,48]. These deleterious organisms tend to be invasive and sporadic, but when they dominate, their harmful effects can overpower any benefits of the usual community.

### (c) Threats to bee microbiomes

The gut microbiota is key to honeybee health, but it is subject to disruption. For example, the gut community composition is drastically affected by exposure to antibiotics [29,49] or to some herbicides [28,30,50]. Bee gut communities can be impacted by diet, including nutrient quantity, nutrient composition and phytochemicals present in nectar, pollen and propolis [51], and by temperature [52,53]. In honeybees, perturbed gut communities are more susceptible to invasion by pathogens, as observed in challenge experiments with *S. marcescens* [28] and *Nosema* [10,54]*.* Honeybee workers with dysbiosis, defined as unhealthy disruption of the normal microbiota, have higher mortality rates within hives [28,29]. Bumblebees also are subject to gut dysbiosis [27,55], which can increase their susceptibility to pathogens [21].

## 3. Approaches and challenges for probiotic treatments

The mounting evidence that the native microbiota is key to bee health but vulnerable to disruption raises the question of whether managed colonies of these insects might be strengthened through probiotic treatments that deliver beneficial bacteria. Such treatments might prevent, or cure dysbiosis. One type of probiotic consists of bacteria that are intended to promote health and a stable microbiome without themselves persisting in the host [1]. Much of the human probiotic industry is based on ingesting organisms such as *Lactobacillus* or *Bifidobacterium* used in fermenting food; these do not colonize guts but may still promote a healthy microbiome although evidence for this is mixed [56].

A second probiotic strategy involves inoculation with microbial strains that are themselves native to the host gut and that could establish and persist long term in the host. For example, hosts experiencing dysbiosis can be treated through the direct transfer of native microbial communities from healthy individuals. In humans, such treatments, called faecal microbiota transplants, have shown efficacy for the treatment of some bowel disorders.

Both approaches are potentially beneficial in bees. Most commercial probiotics currently contain only non-native bacteria from the food industry as in human probiotic mixtures; however, some incorporate a mixture of non-native and native bacteria [57]. Published analyses of persistence in bee guts are not available. However, experimental work has shown that transfers of native gut bacteria between worker honeybees, accomplished by providing homogenate from donor guts to recipient bees, result in stable colonization, typical community composition and host benefits (e.g. [15]). However, direct transfers between hosts carry the risk of introducing pathogenic organisms, potentially causing more harm than benefit, as occasionally observed in human faecal transfers (e.g. [58]).

Below we summarize studies of bee probiotics (electronic supplementary material, table S1). A recent publication provides an exhaustive compilation [57].

### (a) Probiotics for larvae, queen and hive health

The most evident bee pathogens affect larvae, a vulnerable stage of development for honeybees and the stage most visible to beekeepers, since affected bees die in the hive. Larval parasites and pathogens include *Varroa* mites, the bacteria *P. larvae* and *M. plutonius* [43], the chalkbrood fungus *As. apis* and several RNA viruses.

Several investigators have sought to directly protect larvae with probiotics. A strain of *Bombella apis* enhanced larval survival *in vitro* and was investigated as a probiotic hive supplement, delivered in custom-made pollen patties [59]. However, *Bombella apis* had no effect on colony-level measures of brood area, food storage or foraging rate. Bees from hives supplemented with *Bombella apis* appeared to resist *Nosema* infection better, as fewer *Nosema* spores were present after an *in vitro* challenge. Later tests of whether *Bombella apis* inoculation could protect larvae against *M. plutonius* did not reveal protective effects [60]. Miller *et al.* [61] recently demonstrated that some *Bombella apis* strains produce a metabolite that inhibits fungal growth both *in vitro* and *in vivo*. *Nosema* is a microsporidian, a group closely related to fungi, so potentially the same mechanism underlies this fungal suppression and the reduced *Nosema* loads in treated colonies. These experiments were performed in the laboratory, so it remains to be tested if providing these strains as probiotics to hives will be beneficial.

Recently, researchers have also tested commercially available probiotic formulations at the hive scale. Daisley *et al*. [62] focused on hive supplementation with a mixture of two non-bee-associated *Lactobacillus* strains (*Lactobacillus plantarum* and *Lactobacillus rhamnosus*) and one hive-associated strain: *Apilactobacillus kunkeei*. They found that supplementation helped hives resist *P. larvae*. By contrast, Stephan *et al.* [63] provided a probiotic mixture of several hive- and gut-associated strains of *Lactobacillus* and *Bifidobacterium* to hives infected with *P. larvae* and treated or not with antibiotics, but found no improvements in colony fitness. Differences in the design and execution of these experiments, or in the status of the study hives, can explain the different outcomes. These two studies illustrate the persistent issues of reproducibility that face honeybee probiotics research.

Queens remain in the hive and are vital to hive health, and queen longevity and productivity have declined recently, potentially owing in part to hive treatments for mites [64]. The queen's own gut microbiome may influence her health and productivity, but studies of queen microbiomes have not addressed links to fecundity or colony productivity [32,34]. Some probiotic applications appear to improve egg-laying in hives [65,66], potentially owing to impacts on the queen microbiome. Further work is needed to determine if microbiota manipulations in queens can improve hive health.

### (b) Probiotics for adult workers

Workers comprise the vast majority of individuals in hives and are responsible for all hive-level functions except for egg-laying. Colony declines, particularly those associated with classic colony collapse disorder, are often associated with the disappearance of workers from a colony [67]. Since sick workers tend to leave the hive to avoid the spread of disease [68,69], they are less obvious to beekeepers than are sick larvae. Thus, opportunistic pathogens of adult workers are probably underappreciated as factors in overall colony health [48].

Many recent studies indicate that the native worker gut microbiota is perturbed by agrochemicals, such as antibiotics that are used in hives to treat larval infections and in fields to control crop pathogens, and other pesticides, sprayed in areas near colonies. These perturbations are linked to increased susceptibility of workers to infections by opportunistic pathogens [28,29,31,50,54]. For example, workers are more likely to die from *S. marcescens* strains present in hives following microbiota perturbation by antibiotics or glyphosate [28,47]. Potentially, probiotic treatment with natural gut strains from the native microbiota could replenish perturbed gut communities. Recent experimental studies have shown that bees mono-colonized with single or multiple strains of native gut bacteria can control the overproliferation of *S. marcescens* in the bee gut [11,70]. Similar overproliferation is observed in microbiota-deprived bees or antibiotic-treated bees [70].

To date, almost all studies on the use of probiotics to control infections in workers have investigated non-native strains [71,72] In one such study, Daisley *et al*. [62] saw shifts in microbial composition in the guts of worker bees and found that probiotic supplementation, besides increasing the expression of bee immunity genes, such as *defensin-1*, had a negative correlation with the abundance in guts of *Commensalibacter*, *Frischella* and *P. larvae*, but did not change overall bacterial loads. A follow-up study [73] reported that antibiotic-induced dysbiosis in workers could be countered by providing the same probiotic mixture to hives, and that the probiotic bacterial strains were detected in workers post-supplementation.

Native gut probiotics, consisting of isolates from bee guts, could potentially replenish perturbed gut communities and provide sustained protection against pathogens and parasites of workers. Powell *et al*. [31] found that hive treatment with recommended levels of tylosin (antibiotic used against *P. larvae*) results in severe disruption of worker gut communities and in higher mortality when challenged with *S. marcescens*. This increased susceptibility was lessened by treatment with a probiotic mixture of strains of native gut bacteria, suggesting that native gut probiotics can replenish perturbed worker gut communities and thereby reduce pathogenic infections.

*Nosema* also takes advantage of microbial perturbations induced by antibiotics to cause disease in adult workers [54] and can itself perturb the native worker microbiota [74–76]. Many recent probiotic studies in adult honeybees have focused on *Nosema* and have supplemented caged bees or hives with mixtures of bacteria originating from sources other than the native bee microbiota (see the electronic supplementary material, table S1). While some of these experiments reported beneficial effects, such as reduction in *Nosema* spore counts and/or higher survival rates of bees [71,72,77–79], others reported a completely opposite outcome, with increases in spore counts and/or lower survival rates of bees after supplementation [80–82]. A few studies have investigated the effects of probiotics in the control of *Varroa*, showing a reduction in *Varroa* in supplemented hives [66,72,83]. However, these studies did not investigate whether these non-native bacteria persist in the bee gut or affect the native microbiota. To date, clear evidence that probiotics protect workers is lacking.

### (c) What should bee probiotics look like?

Most probiotic efforts in honeybees have not verified the basic mechanisms that contribute to probiotic usefulness. Do probiotic strains establish in bee guts? Also, do probiotics have sustained effects on bee physiology and immune responses?

Native probiotic mixtures should be standardized to contain only a beneficial community, eliminating the risk of introducing harmful entities while including the necessary community diversity to restore a stable, healthy community. Powell *et al*. [31] provided preliminary evidence that a defined community consisting of native gut bacteria can help to ameliorate microbiota perturbation and pathogen susceptibility.

## 4. New experimental results

We performed experiments aimed at addressing questions regarding probiotics for worker gut communities. We tested a defined community of native gut bacteria to examine the ability to establish and persist in the bee gut, comparing this to a commercial probiotic currently in use in apiculture. We also examined the ability of this defined community to stimulate changes in gene expression that are typically induced by the native gut community.

### (a) Natural gut bacteria, but not commercial probiotics, robustly colonize bee guts

To explore the ability of probiotic strains to persist in the bee gut, we placed newly emerged adults directly in sterile cup cages with sugar syrup and sterile pollen. We divided workers into four treatments (approx. 15 bees cup^−1^): no microbial exposure (NP, no probiotic), inoculation with a commercial probiotic (ProB), inoculation with a defined community of co-cultured bee gut microbiota members (DC) or inoculation with a mixture of commercial probiotic with the defined community (ProB + DC). After 5 days, we extracted total gut RNA to produce complementary DNA; thus, dead cells in the gut do not contribute to our assays. We estimated the total load of metabolically active bacteria using quantitative polymerase chain reaction (qPCR) and the taxonomic composition using sequencing of 16S ribosomal RNA (rRNA) amplicons. Detailed methods are included in the electronic supplementary material, methods.

**Figure 2.**
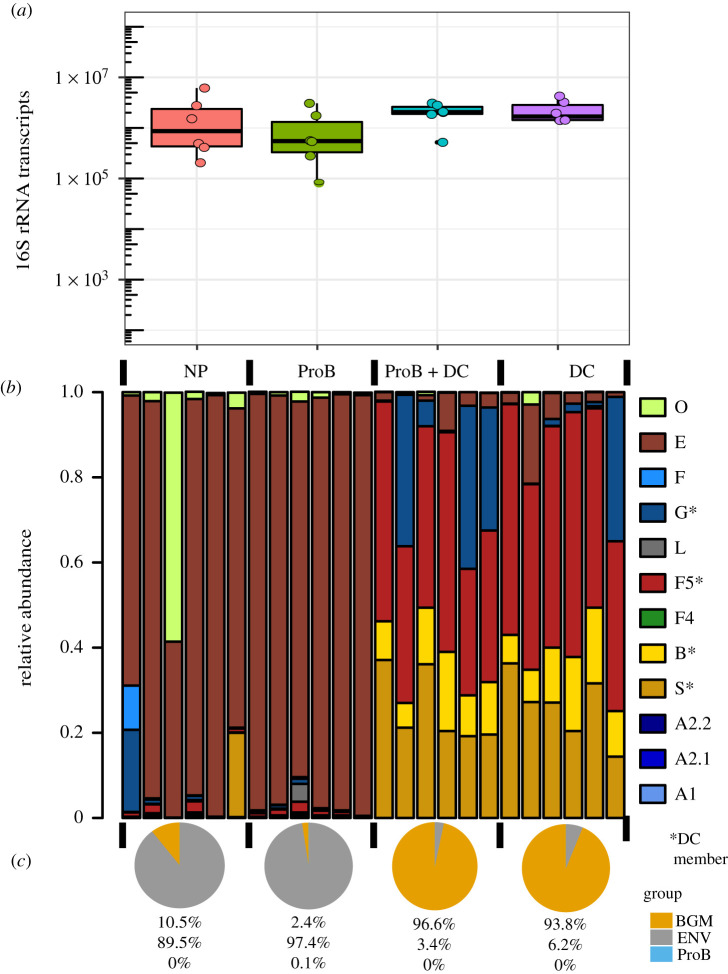
Establishment of commercial probiotic bacteria versus native bee gut bacteria 5 days post-inoculation. (*a*) Total bacterial load estimated by qPCR as 16S rRNA copies per 100 ng RNA. Abbreviations: NP, no probiotic; ProB, probiotic; DC, defined community of native bee gut bacteria; or ProB + DC, mixture of ProB plus DC. All conditions have similar bacterial loads (*p* > 0.05, Kruskal–Wallis). (*b*) Relative abundances of bacterial lineages as determined by 16S V4 sequencing. Abbreviations: A1, *Bartonella apis*; A2.1, *Commensalibacter* spp.; A2.2, *Bombella apis*; S, *Snodgrassella alvi*; B, *Bifidobacterium* spp.; F4, *Bombilactobacillus* spp.; F5, *Lactobacillus* nr. *melliventris*; L, environmental Lactobacilli; G, *Gilliamella* spp.; F, *Frischella perrara*; E, Enterobacteriaceae; O, other uncategorized. NP and ProB groups are dominated by Enterobacteriaceae, and both groups fed DC are dominated by core bee gut bacteria present in the DC. (*c*) Average percentages of bacterial taxa grouped by origin. BGM, taxa of bee gut microbiota; ENV, environmentally associated taxa; ProB, taxa in commercial probiotic mixture. Data are available online in the electronic supplementary material.

Bees in all of the treatments achieved similar bacterial loads (figure 2*a*). The presence of high levels of bacteria in the NP group suggests inoculation from frame surfaces as the young bees emerged, as previously observed [8]. Communities from bees in the NP group and the ProB group have a very similar taxonomic composition (figure 2*b*). Both groups had large components of Enterobacteriaceae, including genera that are opportunistic pathogens. An evaluation of bacteria by probable source (environment/commercial probiotic/native bee gut community) showed that the NP and ProB conditions were dominated by environmentally acquired bacteria (figure 2*c*). In the ProB treatment, the component probiotic taxa were present at less than 1% on average. Thus, bacteria from a non-bee gut origin establish poorly in the gut, enabling increases in opportunistic environmentally acquired taxa, largely Enterobacteriaceae. By contrast, in both treatments where native gut bacteria were used (ProB + DC and DC), native gut strains dominated (greater than 93% on average) and limited proliferation of environmentally acquired bacteria. Native strains dominated at 5 days when bees were harvested and the experiment terminated. Previous experiments showed persistence for at least 10 days following inoculation of microbiota-free bees with bee gut homogenates and further showed that the characteristic bee gut microbiota persists for the life of the adult worker under natural conditions [3,8,9]. Thus, the DC community is probably stable for longer periods.

### (b) Defined native probiotics mimic effects of natural communities on host gene expression

Natural gut communities have been shown to modulate the expression of honeybee genes, including key genes involved in immunity, metabolism and development, and bees lacking a normal gut community have abnormal immune function, metabolism and weight gain [10,11,15,16]. We thus investigated if a defined community of native gut bacteria resembles complex natural gut communities in effects on host gene expression.

Late-stage pupae were removed from a frame and placed in plastic chambers in incubators as in [8], allowed to eclose as adults, then placed in cup cages with sugar syrup and sterile pollen. Previous work shows that this yields bees devoid or nearly devoid of gut bacteria [3,8]. We divided bees into three sets. One set (MD, microbiota deprived) was not exposed to bacteria, one set (DC, defined community of native strains) was fed a mixture of native gut strains, and one set (GH, gut homogenate) was fed fresh gut homogenate from hive workers, containing the full natural gut community. We measured expression of genes previously shown to be induced by colonization with the full gut community, including genes involved in development [15] and immunity [11,23]. In one experiment, we assayed genes for several antimicrobial peptides (apidaecin, abaecin, defensin and hymenoptaecin) known to be expressed in specific tissues. In a second experiment, we assayed genes involved in hormonal signalling (vitellogenin, insulin receptors and insulin-like peptides) and expected to be expressed throughout the body. We also assayed bacterial loads using qPCR.

**Figure 3.**
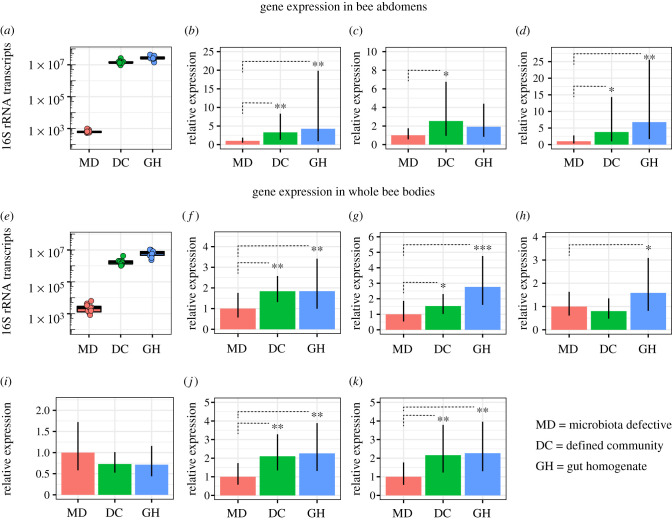
Expression of genes underlying immunity and hormonal signalling in bees lacking a native microbiota (MD), colonized with a defined community of native bee gut strains (DC), or colonized with gut homogenate from a donor bee (GH). (*a*) Bacterial load estimated as bacterial 16S rRNA copies and (*b–d*) relative transcript levels of genes encoding abaecin (*b*), apidaecin (*c*) and hymenoptaecin (*d*) in bee abdomens across groups (*n* = 11 per group). (*e*) Bacterial 16S rRNA copies and (*f–k*) relative transcript levels of genes encoding defensin 2 (*f*), vitellogenin (*g*), insulin-like peptides 1 (*h*) and 2 (*i*), and insulin receptors 1 (*j*) and 2 (*k*) in whole bee bodies across groups (*n* = 10 per group). The linear regression ‘lm’ option in the pcr package in R was applied to estimate differences between groups. ***p < 0.001, **p < 0.01, *p < 0.05. Data are available online in the electronic supplementary material. (Online version in colour.)

DC and GH bees achieved similar bacterial loads, which were far higher than those of MD bees (figure 3*a*,*e*). The higher loads correspond to pronounced activation of key bee genes in abdomen samples (higher expression of genes encoding abaecin, apidaecin and hymenoptaecin in DC and GH bees than in MD bees) (figure 3*b–d*) and in whole-body samples (higher expression of genes encoding defensin-2, vitellogenin, and insulin receptors 1 and 2 in DC and GH bees, and insulin-like peptide 1 in GH bees) (figure 3*f–k*). Thus, the defined community mimics the native full microbiota in inducing the expression of bee genes involved in immunity and metabolism.

## 5. Engineered probiotics in bees

Native gut bacteria could also be genetically engineered to better enhance bee health. Synthetic biologists have engineered probiotic bacteria to perform additional functions and express new traits, and these engineered probiotics represent the newest frontier for manipulating microbiomes. Because bee gut bacteria can be grown in the laboratory and genetically manipulated, researchers have begun to create and test engineered probiotics [84–86]. Engineered bee probiotics could be enhanced to perform a new function (degradation of pesticides or other harmful xenobiotic compounds), gain resistance to a specific stressor (resistance to pesticides), or even manipulate bee behaviour and immunity. Engineered strains would then be reintroduced into honeybees and hives where they would coexist with natural strains and perform their desired function. This approach mirrors recent efforts to engineer the human microbiome by modifying commensal bacteria from human guts to fight pathogens and treat disease [87–89], and similar efforts are underway to modify gut communities associated with other insects [90], plants and other animals [91].

While microbiome engineering to improve bee health remains in its infancy, a recent study demonstrated the feasibility of this approach [92]. In this study, researchers engineered the symbiotic bee gut bacterium *S. alvi* to express double-stranded RNA in the gut of honeybees which then triggers the bee RNA-interference immune response. In laboratory experiments, this approach successfully altered bee gene expression and limited damage from deformed wing virus and *Varroa* mites.

The feasibility and effectiveness of the approach has not been tested under field conditions, and it is not yet known if engineered strains would persist in an actual hive, and whether genetic constructs would stably function or need to be periodically reintroduced. Another unknown is how engineered strains might affect the natural ecosystem and bacterial community in bees. Appropriate risk assessments would be required to assess potential for negative impact. While no engineered bacteria have been used in bumblebees or other social bees, similar approaches to those used in honeybees are plausible.

## 6. Future prospects for honeybee probiotics

Despite efforts to develop honeybee probiotics, no probiotic formulations have been demonstrated to be reliably effective in honeybees. Currently available probiotic formulations for bees include strains of *Lactobacillus* and *Bifidobacterium* used in fermenting dairy products and incorporated into human probiotic formulations; these are foreign to bee guts. Results presented here, and general surveys of bee gut microbiota, indicate that these bacteria do not establish to high titers or persist in bee guts. Even *Apilactobacillus* strains that are associated with diverse bees, honey or nectar, may not stably colonize guts of *Apis* or *Bombus* species. Potentially, probiotics can provide benefits without establishing in hosts, but robust evidence is not available. Ingesting substantial quantities of live bacteria that do not occur naturally in hosts has the potential to do harm, so rigorous experiments that evaluate effects are needed.

We found that naturally occurring strains of bee gut bacteria can be administered as a defined community to bees where they re-establish and persist, whereas a commercial probiotic formulation composed of non-native bacteria does not establish in bee hosts (figure 2). Furthermore, the communities established by native strains resemble natural bee gut communities in composition and in their activation of bee genes related to immunity and development (figure 3). These results are consistent with preliminary trials showing that the administration of natural isolates can restore microbiota disrupted by antibiotics and can defend against infection by opportunistic bacterial pathogens [31,70]. Although promising, establishment does not imply efficacy in promoting host health, and the benefits of native bee gut strains remain untested at the hive level. The use of natural gut isolate strains would also require methods to grow these bacteria at scale, combine them into communities and deliver them to hives. Since even closely related strains may vary in their metabolic capabilities, and related bee gut strains vary in responses to chemical stressors such as antibiotics and glyphosate, it also would be useful to identify the specific strains that maximize benefits to bees.

Though some questions are unanswered, the future of probiotics for honeybees is bright. It may be possible to design specific communities of natural gut isolates that stably replenish gut communities disrupted by the many stressors bees face and that are economical and efficient for use in apiaries.

## Data Availability

Data for figures 2 and 3 are provided in the electronic supplementary material [93].
